# Squeal from the Eclectics

**Published:** 1893-01

**Authors:** 


### *EDITORIAL.*

The office of THE JOURNAL is in room 330 Equitable Building.
Address communications relating to the Editorial Department to DR. L. B. GRANDY, Atlanta, Ga., Box 431.
Business communications should be addressed to DR. M. B. HUTCHINS, Atlanta, Ga.
All remittances should be made payable to "ATLANTA MEDICAL AND SURGICAL JOURNAL."
Articles for publication should reach this office not later than the 15th instant.
The editors are not responsible for views expressed by contributors.

### A SQUEAL FROM THE ECLECTICS.

Possibly the highest tribute that the Examiners' Bill has received is the opposition which it encountered from the eclectic sect, *et id omne genus*. In spite of an honest effort to be fair to these latter on the part of the gentlemen who framed the bill, it did not fail to receive their disapproval. We like it for the enemies it has made. But here is the eclectic wail delivered (Eclectic Medical *Journal*, December) by the same eclectic gentleman, whose "fame is national as surgeon, and in the treatment of chronic disease" (see daily press):

MEDICAL LEGISLATION IN GEORGIA.—We are gratified to announce that the unjust and infamous bill known as Senate bill No. 51, entitled an act to establish a board of medical examiners for the State of Georgia, and which accidentally passed the Senate through the neglect of those who would have been present and defeated it had they known the iniquity of the bill—the committee of the House moved to lay it on the table till next session of the Legislature. We predict that the present bill will never be resurrected again. Not only are the majority of the doctors in the State, but the people of the State are aroused to an extent never before known in Georgia in opposition to such an infamous measure. The whole thing was concocted in the brains of a certain clique of Atlanta doctors, who are conceited enough to believe that all medical wisdom in Georgia is concentrated in their craniums. So iniquitous is said Senate bill No. 51, that, if by chance it should become a law, it would be met with open defiance by a majority of the physicians and people of the State; and we honestly believe that Governor Northen would veto such a measure. Let the doctors of all schools agree upon a just, reasonable bill, one that would be fair to all schools of medicine, then it may be possible to change the present law of Georgia regulating the practice of medicine, otherwise we put the scheming Atlanta clique of doctors on notice that they will meet with opposition greater than that before the House committee.

So much from the same high eclectic source which cried about one year ago that "impostors are infesting the State and quackery is rampant in Georgia." Indeed it seems that the jewel of consistency does not ornament the features of the Georgia Eclectic Medical *Journal* and its famous editor.

---

MR. GREGG, of the firm of Beck & Gregg, who are advertising Victor bicycles in THE JOURNAL, has shown us a letter of inquiry from Philadelphia and one from Texas, stating that they saw the advertisement in THE ATLANTA MEDICAL AND SURGICAL JOURNAL. How is this for an advertising medium ?

---

IN a friendly manner we wish to call the attention of the editor of the (New York) *Journal of Cutaneous and Gunito-Urinary Diseases* to the numerous typographical errors appearing in his publication—notably the December number. We would suggest a "stirring up" of the proof-reader.

